# Legacy Effects of Phytoremediation on Plant-Associated Prokaryotic Communities in Remediated Subarctic Soil Historically Contaminated with Petroleum Hydrocarbons

**DOI:** 10.1128/spectrum.04448-22

**Published:** 2023-03-28

**Authors:** Jakub Papik, Michal Strejcek, Lucie Musilova, Rodney Guritz, Mary-Cathrine Leewis, Mary Beth Leigh, Ondrej Uhlik

**Affiliations:** a University of Chemistry, and Technology, Prague, Faculty of Food and Biochemical Technology, Department of Biochemistry and Microbiology, Prague, Czech Republic; b Institute of Arctic Biology, University of Alaska Fairbanks, Fairbanks, Alaska, USA; c Department of Biology and Wildlife, University of Alaska Fairbanks, Fairbanks, Alaska, USA; d Agriculture and Agri-Food Canada, Quebec, Quebec, Canada; Istituto Italiano di Tecnologia

**Keywords:** bacterial communities, endophyte, petroleum hydrocarbons, phytoremediation, rhizosphere, secondary succession, subarctic, rhizosphere-inhabiting microbes

## Abstract

Phytoremediation of petroleum hydrocarbons in subarctic regions relies on the successful establishment of plants that stimulate petroleum-degrading microorganisms, which can be challenging due to the extreme climate, limited nutrients, and difficulties in maintaining sites in remote locations. A long-term phytoremediation experiment was initiated in Alaska in 1995 with the introduction of grasses and/or fertilizer to petroleum hydrocarbon (PHC)-contaminated soils that were subsequently left unmanaged. In 2011, the PHC concentrations were below detection limits in all soils tested and the originally planted grasses had been replaced by volunteer plant species that had colonized the site. Here, we sought to understand how the original treatments influenced the structure of prokaryotic communities associated with plant species that colonized the soils and to assess the interactions between the rhizospheric and endophytic communities of the colonizing vegetation 20 years after the experiment was established. Metataxonomic analysis performed using 16S rRNA gene sequencing revealed that the original type of contaminated soil and phytoremediation strategy influenced the structure of both rhizospheric and endophytic communities of colonizing plants, even 20 years after the treatments were applied and following the disappearance of the originally planted grasses. Our findings demonstrate that the choice of initial phytoremediation strategy drove the succession of microorganisms associated with the colonizing vegetation. The outcome of this study provides new insight into the establishment of plant-associated microbial communities during secondary succession of subarctic areas previously contaminated by PHCs and indicates that the strategies for restoring these ecosystems influence the plant-associated microbiota in the long term.

**IMPORTANCE** Subarctic ecosystems provide key services to local communities, yet they are threatened by pollution caused by spills and disposal of petroleum waste. Finding solutions for the remediation and restoration of subarctic soils is valuable for reasons related to human and ecosystem health, as well as environmental justice. This study provides novel insight into the long-term succession of soil and plant-associated microbiota in subarctic soils that had been historically contaminated with different sources of PHCs and subjected to distinct phytoremediation strategies. We provide evidence that even after the successful removal of PHCs and the occurrence of secondary succession, the fingerprint of the original source of contamination and the initial choice of remediation strategy can be detected as a microbial legacy in the rhizosphere, roots, and shoots of volunteer vegetation even 2 decades after the contamination had occurred. Such information needs to be borne in mind when designing and applying restoration approaches for PHC-contaminated soils in subarctic ecosystems.

## INTRODUCTION

Subarctic regions are often impacted by fuel and oil spills associated with petroleum extraction, transportation, storage, and use (1–5). This release of petroleum hydrocarbons (PHCs) into soils and/or water can pose exposure risk to humans, impair environmental health, and disrupt diverse microbial communities and their ecological functions in the environment (6, 7). Since natural attenuation of contaminants proceeds more slowly in cold regions than in warmer areas, it is not always a suitable approach for their restoration (8). Therefore, the development of appropriate remediation strategies is of great environmental significance in these regions.

Phytoremediation, the use of plants and plant-associated microorganisms to remediate contaminated areas, can be a cost-effective and sustainable strategy for the ecological restoration of PHC-polluted sites (9, 10). Unlike commonly used physical and chemical remediation strategies, phytoremediation does not necessarily cause further disturbance of the landscape and can help re-establish vegetation in the ecosystem (11). To date, there have been a limited number of studies examining the phytoremediation of PHC-contaminated sites at high latitudes (12–16). The short growing season, low precipitation, and nutrient limitation due to slow chemical weathering and biological decomposition dramatically limit the productivity of plants and their associated microbes in some cold regions (8, 17). Moreover, high concentrations of PHCs can impair the water movement in soil and gas exchange between soil and air. Such processes can restrict plant growth and lower the activity of soil microorganisms, thereby decreasing soil health (18–20). Therefore, identifying plant species that can thrive under these extreme conditions and successfully remediate PHC-contaminated soils is important to the development of successful phytoremediation strategies for subarctic regions.

(Re)introduction of plants to contaminated areas has been shown to promote ecosystem recovery by increasing soil health through the improvement of soil structure and the accumulation of organic carbon and limiting nutrients, such as nitrogen or phosphorus (21, 22). Microorganisms living in the rhizosphere and endosphere play important roles in these processes (23–25). Both the rhizosphere, the narrow zone of soil directly influenced by the roots, and the endosphere, the plant interior, have been shown to harbor bacteria that often stimulate plant growth through fixation of atmospheric nitrogen, solubilization of inorganic phosphate, or production of phytohormones and siderophores or help to protect their host plants by warding off phytopathogens and herbivores (25, 26). In addition, some endophytes have been found to mitigate the impacts of contaminant-associated stress by directly degrading pollutants (27, 28). Overall, plant-associated bacteria enable their hosts to adapt to changing environmental conditions, and their beneficial properties could potentially be even more important under the harsh conditions that prevail in subarctic regions (29, 30). While the composition of plant-associated bacterial communities is generally influenced by a variety of factors, such as plant species, soil characteristics, or climate (25), the exact mechanisms that drive the assembly of plant microbiota are likely to be a combination of factors and may vary across different environments.

**FIG 1:**
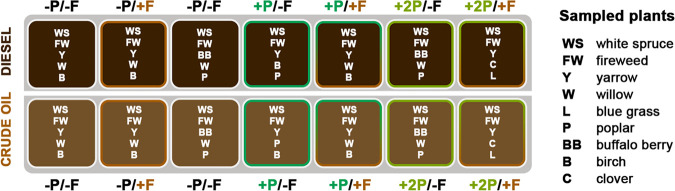
The design of the experimental phytoremediation site. Fourteen treatment plots were established in 1995; half of the soils were contaminated with diesel fuel (top) and half with crude oil (bottom). Plots were sown with annual ryegrass (+P) or a mixture of annual ryegrass and Arctared fescue (+2P) or left unplanted (−P) and were treated with commercial mineral fertilizer (+F) or left unfertilized (−F). In 2014, plant species that had colonized the treatment plots (WS, FW, Y, W, L, P, BB, B, and C) were harvested; five individual plants belonging to different species were collected from each plot.

In this study, we built upon a long-term phytoremediation experiment with PHC-contaminated soils established in Fairbanks, Alaska, in 1995 (3, 31–33). The original study determined how the introduction of different grass species, fertilization, or their combination would affect the remediation of PHC-contaminated soils over time. We re-examined the field site after it had experienced no active site management for almost 20 years. By that time, the PHC concentrations were below detection limits in all soils and the originally planted grasses had already disappeared and had been replaced by volunteer vegetation depending on the originally applied phytoremediation strategy (12). Here, we hypothesized that the legacy of previously applied bioremediation strategies would still be detectable in microbial communities associated with volunteer vegetation (Fig. 1). Together, our investigations of this research site have helped to uncover how different factors contribute to both plant and microbial secondary succession in PHC-contaminated subarctic environments.

## RESULTS

### Diversity and structure of plant-associated prokaryotic communities.

**FIG 2:**
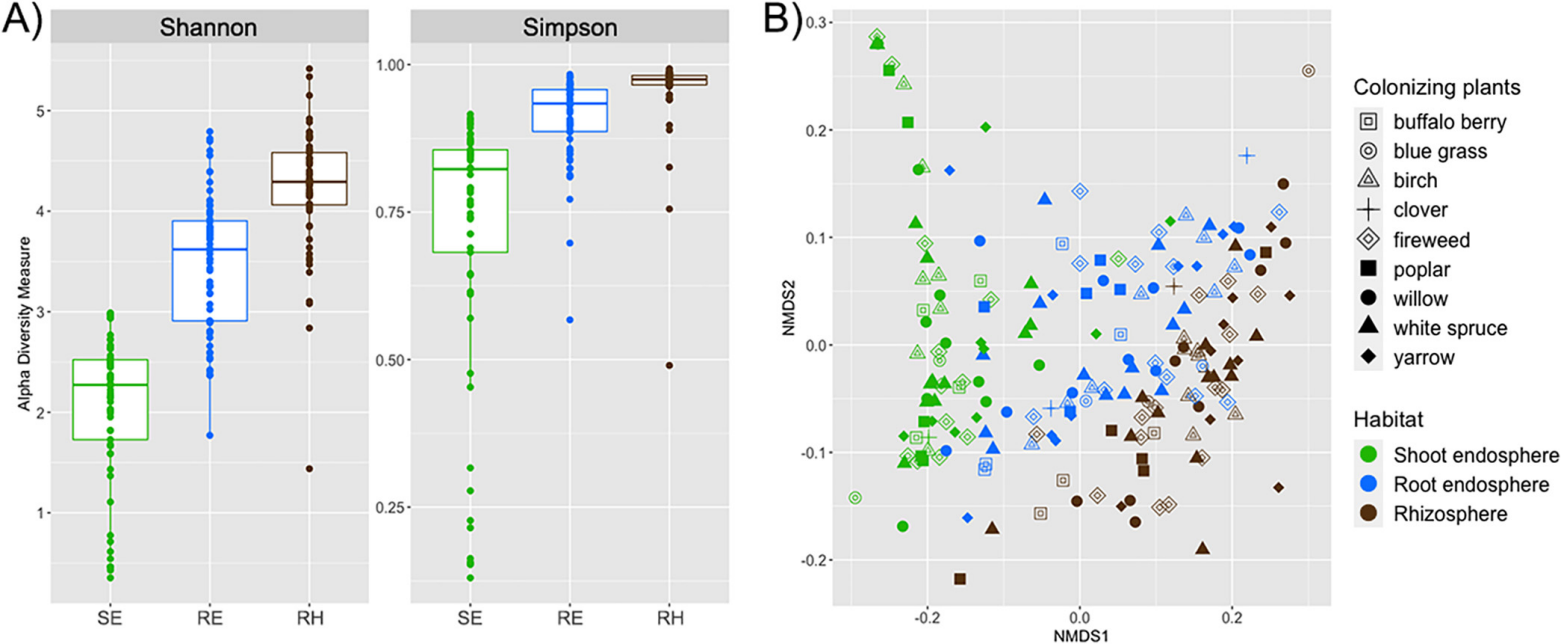
(A) Shannon and Simpson alpha diversity indices calculated from 16S rRNA gene ASVs originating from the shoot endosphere (SE), root endosphere (RE), and rhizosphere soil (RH) samples of colonizing plants. (B) Nonmetric multidimensional scaling (NMDS) using weighted UniFrac distances of prokaryotic communities in the rhizosphere and root and shoot endosphere samples of colonizing plants. The significant effect of plant-associated habitat (shoot endosphere, root endosphere, and rhizosphere) on the structure of prokaryotic communities was confirmed by PERMANOVA (*P* = 0.001).

The prokaryotic diversities in the rhizosphere and both the root and shoot endospheres of colonizing plants (Picea glauca [white spruce; WS], Epilobium angustifolium [fireweed; FW], Achillea millefolium [yarrow; Y], Salix spp. [willow; W], Poa spp. [blue grass; L], Populus balsamifera [poplar; P], Shepherdia canadensis [buffalo berry; BB], Betula neoalaskana [birch; B], and Trifolium hybridum [clover; C]) were assessed by Shannon and Simpson alpha diversity indices (Fig. 2A). Both Shannon and Simpson indices differed significantly among the plant-associated microbial habitats studied (*P ≤ *0.001, Kruskal-Wallis test); the prokaryotic diversity decreased in the order of rhizosphere, root endosphere, shoot endosphere. Nonmetric multidimensional scaling (NMDS) using weighted UniFrac distances revealed that the communities in the rhizosphere and shoot endosphere clustered separately (Fig. 2B), indicating that rhizospheric communities differed from those found in the shoots, while the communities in the roots were more similar to both rhizospheric and shoot endophytic communities. There was no clear separation based on the colonizing plant species, indicating that communities hosted by different species were not necessarily dissimilar (Fig. 2B).

### Association of prokaryotic communities with the original remediation strategies.

The assessment of prokaryotic diversity in the rhizosphere, root endosphere, and shoot endosphere across different treatments is shown in Fig. S1 in the supplemental material. Out of the studied factors, the original soil type (crude oil contaminated or diesel contaminated) was found to be significantly associated with the prokaryotic diversity (*P ≤ *0.05, Kruskal-Wallis test) in the rhizosphere, while the original phytoremediation strategy significantly influenced the prokaryotic diversity in the root endosphere (*P ≤ *0.05, Kruskal-Wallis test). In the shoot endosphere, both the original soil type and original phytoremediation strategy were significantly associated with prokaryotic diversity (*P ≤ *0.01 and *P ≤ *0.001, respectively, Kruskal-Wallis test). Fertilization was not found to be significantly associated with prokaryotic diversity in any studied habitat (*P > *0.05, Kruskal-Wallis test). Additionally, the interaction between the original type of soil and the phytoremediation strategy was found to be significantly associated with the structure of prokaryotic communities in all three habitats studied: the rhizosphere and root and shoot endospheres (*P ≤ *0.05, permutational multivariate analysis of variance [PERMANOVA]) (Table 1).

**TABLE 1:** The associations of the original soil type, phytoremediation strategy, and fertilization and their interactions with the structures of prokaryotic communities in the rhizosphere and root and shoot endospheres of colonizing host plants

Parameter(s)	Rhizosphere			Root endosphere			Shoot endosphere		
	*R* ^2^	*F*	*P* ^*a*^	*R* ^2^	*F*	*P* ^*a*^	*R* ^2^	*F*	*P* ^*a*^
Original soil type	0.10	7.47	0.001*	0.09	6.64	0.001*	0.23	24.65	0.001*
Phytoremediation strategy	0.04	1.65	0.003*	0.05	1.86	0.003*	0.10	5.59	0.001*
Fertilization	0.03	2.20	0.002*	0.02	1.60	0.056	0.01	0.83	0.489
Original soil type × phytoremediation strategy	0.04	1.54	0.006*	0.05	1.77	0.002*	0.11	5.71	0.001*
Original soil type × fertilization	0.03	2.30	0.001*	0.02	1.59	0.034*	0.01	1.09	0.301
Fertilization × phytoremediation strategy	0.03	1.17	0.110	0.03	1.13	0.194	0.03	1.62	0.129

a Significant *P* values by PERMANOVA (alpha = 0.05) are labeled with an asterisk.

**FIG 3:**
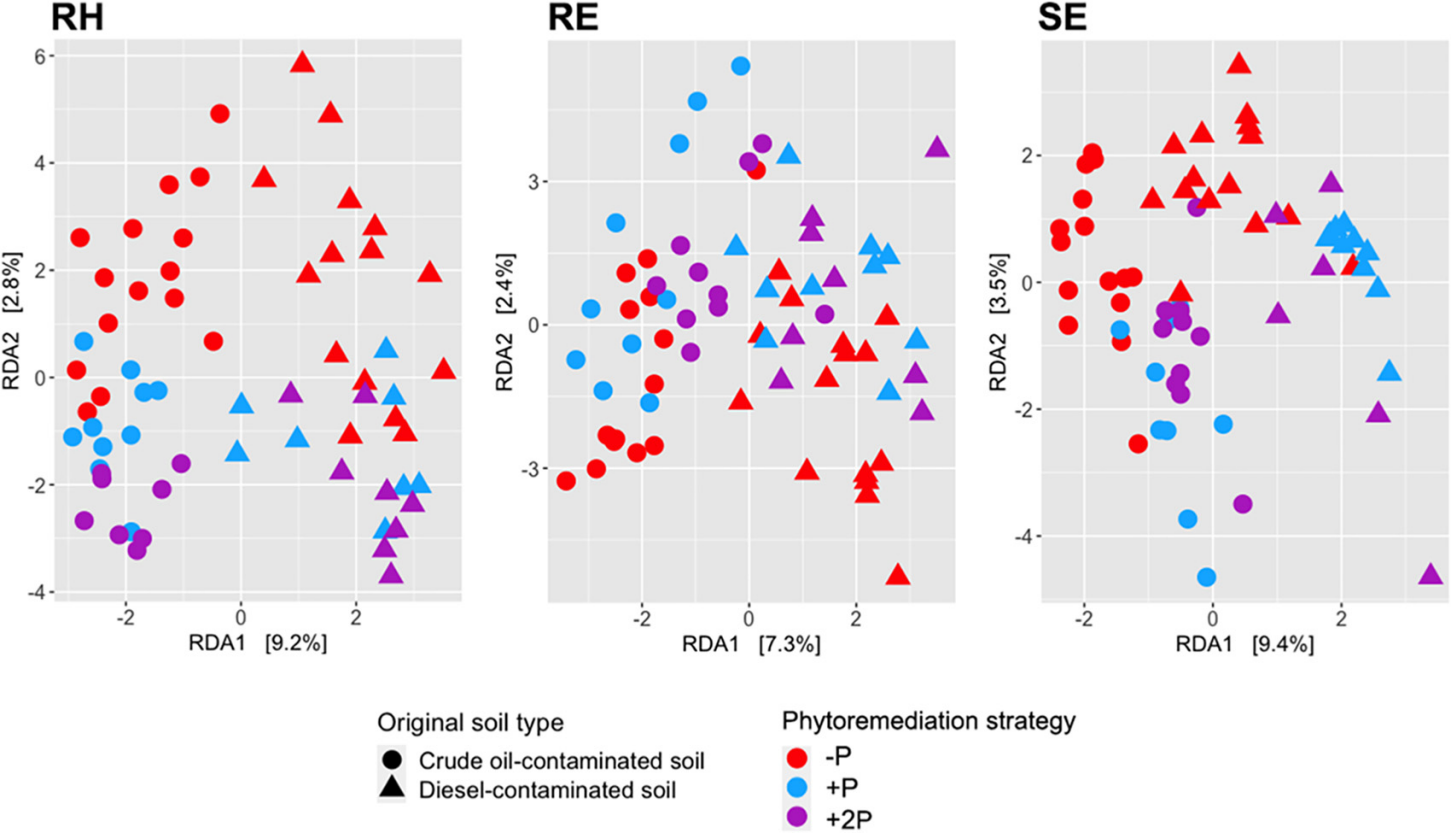
Redundancy analysis (RDA) of centered log ratio-transformed sequence data of prokaryotic communities in the rhizosphere (RH), root endosphere (RE), and shoot endosphere (SE) samples. Phytoremediation strategies included planting with annual ryegrass (+P) or a mix of annual ryegrass and Arctared fescue (+2P) or unplanted control (−P).

The dominant effect of the original soil type on prokaryotic communities in all studied habitats was further demonstrated by redundancy analysis (RDA). Prokaryotic communities in both soil types formed distinct clusters in the ordination space and were separated by the first RDA axis (Fig. 3). Additionally, the rhizospheric soil communities were separated based on the original phytoremediation strategy applied as well (Fig. 3).

**FIG 4:**
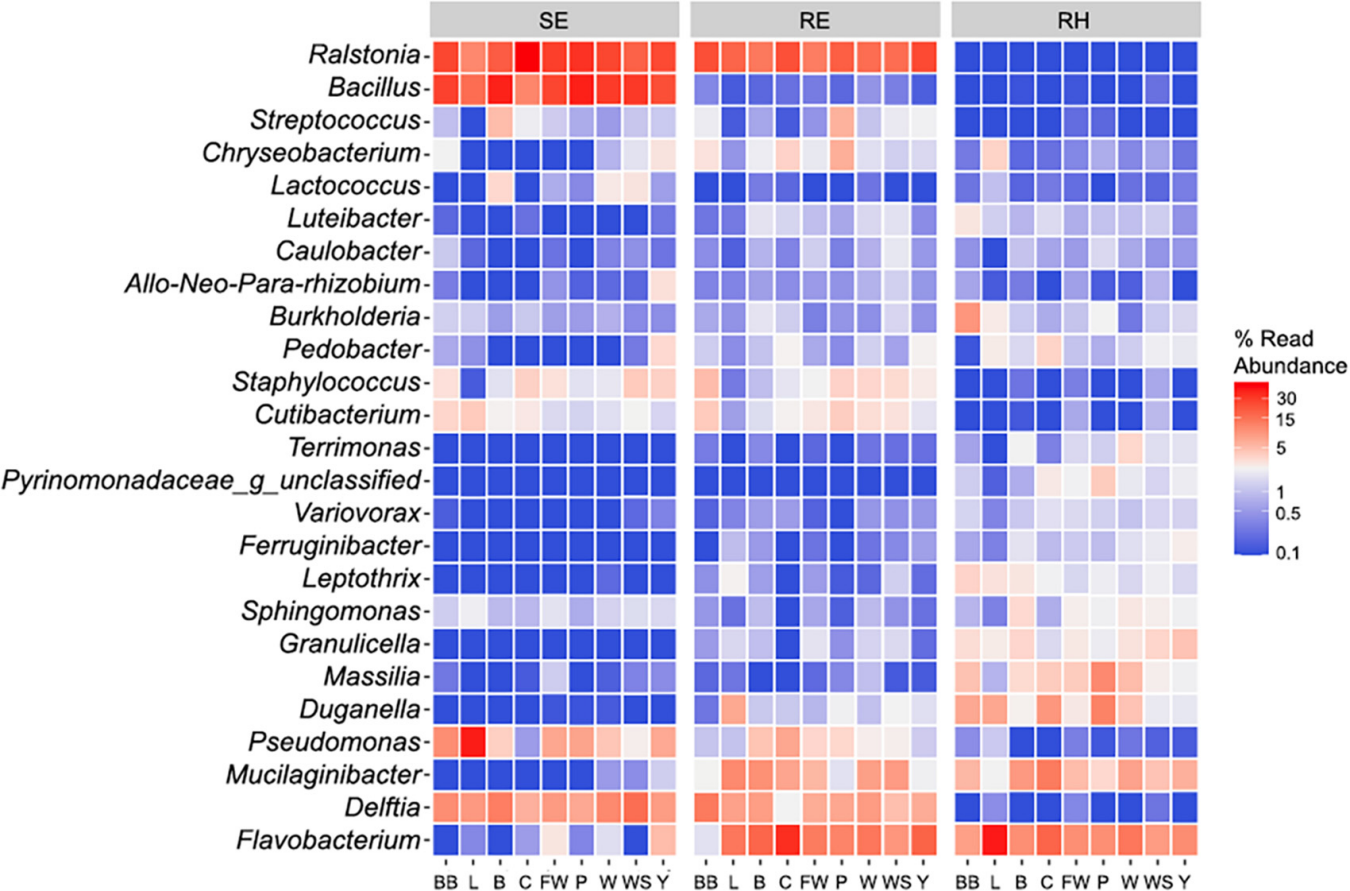
Heat map of the 25 most abundant prokaryotic genera in the rhizosphere (RH), root endosphere (RE), and shoot endosphere (SE) samples of colonizing plant species (BB, L, B, C, FW, P, W, WS, Y).

When included in the PERMANOVA model, the colonizing plant species were not found to be significantly associated with the microbial community structure (tested separately on rhizospheric, root endophytic, and shoot endophytic data sets) (*P > *0.05). It should be noted that since the experimental plots were not uniformly vegetated due to distinct patterns of secondary succession (Fig. 1), we were not able to sample the same number of individuals for all plant species. Thus, the statistical power was likely not the same for all plant species sampled. For that reason, the variable of colonizing plant species was excluded from the final PERMANOVA analysis (Table 1) and the association of colonizing plant species with community structure was investigated directly by analyzing the relative abundances of the top 25 prokaryotic genera in the rhizosphere, roots, and shoots of volunteer vegetation (Fig. 4). The most abundant genera were found to be present in similar relative abundances across plant species (Fig. 4). The relative abundances of the top 25 prokaryotic genera in the rhizosphere, root, and shoot samples across different treatments are shown in Fig. S2.

### Significantly enriched prokaryotic genera in the rhizosphere and endospheres.

**FIG 5:**
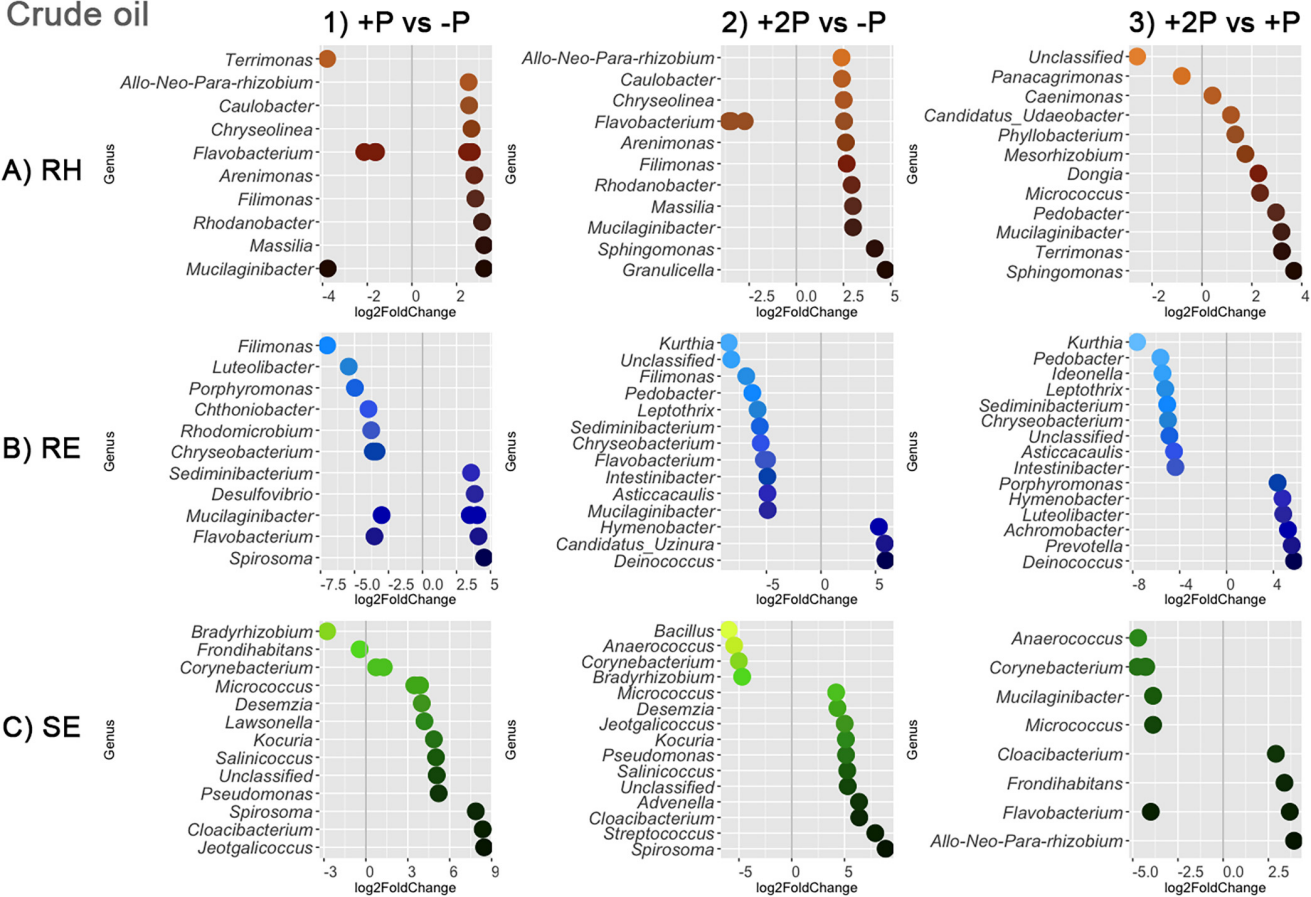
Differential abundance analysis showing significantly enriched (*P*_adj_ < 0.01) prokaryotic ASVs in rhizosphere (RH) (A), root endosphere (RE) (B), and shoot endosphere (SE) (C) samples of colonizing plants growing in soil previously contaminated with crude oil and subjected to different phytoremediation strategies. Pairwise comparisons include (1) previous phytoremediation by Lolium multiflorum (+P) versus control (−P), (2) previous phytoremediation by a combination of Lolium multiflorum and Festuca rubra (+2P) versus −P, and (3) +2P versus +P. Negative log_2_ fold change values represent ASVs significantly enriched in a treatment listed to the left of the vertical gray line, while positive log_2_ fold change values represent ASVs significantly enriched in a treatment listed to the right of the vertical gray line. Only the top 15 differently abundant ASVs with the highest log_2_ fold change values per pairwise comparison are displayed. Multiple log_2_ fold change values per row represent different ASVs belonging to the same genus.

**FIG 6:**
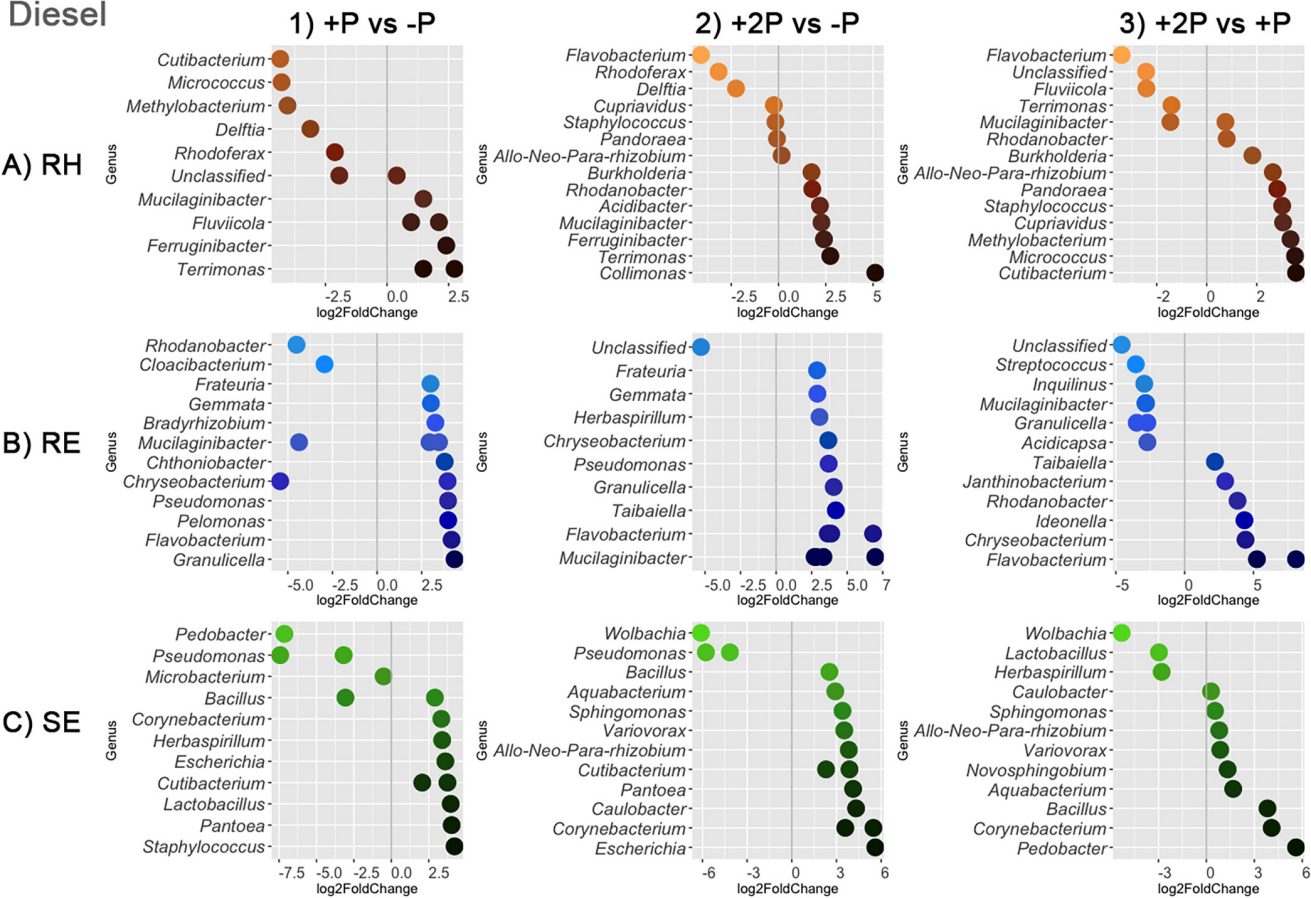
Differential abundance analysis showing significantly enriched (*P*_adj_ < 0.01) prokaryotic ASVs in rhizosphere (RH) (A), root endosphere (RE) (B), and shoot endosphere (SE) (C) samples of colonizing plants growing in soil previously contaminated with diesel and subjected to different phytoremediation strategies. Pairwise comparisons include (1) previous phytoremediation by Lolium multiflorum (+P) versus control (−P), (2) previous phytoremediation by a combination of Lolium multiflorum and Festuca rubra (+2P) versus −P, and (3) +2P versus +P. Negative log_2_ fold change values represent ASVs significantly enriched in a treatment listed to the left of the vertical gray line, while positive log_2_ fold change values represent ASVs significantly enriched in a treatment listed to the right of the vertical gray line. Only the top 15 differently abundant ASVs with the highest log_2_ fold change values per pairwise comparison are displayed. Multiple log_2_ fold change values per row represent different ASVs belonging to the same genus.

To investigate the legacy effects of phytoremediation in more detail, differential abundance analysis was conducted to identify prokaryotic amplicon sequence variants (ASVs) that were significantly enriched (adjusted *P* value [*P*_adj_] of <0.01 [Wald test with Benjamini-Hochberg multiple testing correction]). The samples of crude oil-contaminated soil and diesel-contaminated soil were analyzed separately, as there was a significant interaction between original soil type and phytoremediation strategy (Table 1). For each soil type, three pairwise comparisons were conducted among treatments: (i) planting with one grass species versus the no-remediation control (+P versus −P), (ii) planting with two grass species versus the control (+2P versus −P), and (iii) +P versus +2P. The use of different strategies led to the enrichment of distinct ASVs in both the rhizosphere and endospheres of the plants that succeeded in crude oil-contaminated soil (Fig. 5) and diesel-contaminated soil (Fig. 6).

## DISCUSSION

In this study, we investigated the prokaryotic communities associated with plants that had colonized a phytoremediation study site previously contaminated with PHCs (diesel or crude oil) in subarctic Alaska. The studied soils were originally subjected, in 1995, to different phytoremediation strategies; specifically, they were sown with one or two grass species or were left unplanted (+P, +2P, and −P, respectively), with or without fertilization (−F and +F) (3, 12, 31, 33). The site was subsequently left unmanaged for almost 20 years. In the meantime, both types of soils successfully reached remediation targets, most likely with the help of both the original treatments and colonizing vegetation, which replaced the initial grasses planted on the experimental site and followed different successional trajectories based on the original treatment (12). Here, we aimed to decipher whether and how the original treatments shaped the prokaryotic communities, not only in the rhizosphere but also in the root and shoot endospheres of the colonizing vegetation, 20 years after the original study was established. We found that of the factors studied, the interaction between the original type of contaminated soil and the initial phytoremediation strategy was primarily associated with the structures of the rhizospheric and endophytic prokaryotic communities of the colonizing plants.

Overall, the rhizospheres, root endospheres, and shoot endospheres of the colonizing plants differed from each other in both the structure and diversity of prokaryotic communities, demonstrating an effect of plant physiology on all microbial habitats studied (Fig. 2). Such a finding is in line with previous studies that showed that below-ground and above-ground plant-associated habitats host different microbial communities—for example, Lopez-Echartea et al. (14), Coleman-Derr et al. (34), and Yang et al. (35). More importantly, even after nearly 20 years and the disappearance of the originally planted grasses, not only did the original soil type (diesel- or crude oil-contaminated soil) significantly influence the structures of prokaryotic communities in all studied habitats of colonizing plants, but so did the phytoremediation strategy (−P, +P or +2P) (Table 1; Fig. 3). In fact, there was an interactive effect of these two variables. The observed influence of the original soil type (crude oil- or diesel-contaminated soil) on community structure is not very surprising, as the two experimental soils differed in texture as well as the original source of PHCs. This finding is in accordance with previous studies (36–41), which demonstrate that soil characteristics, together with plant species, are generally among the major factors driving plant-associated communities. To our surprise, we did not find the identity of the host plant to be significantly associated with the structure of the prokaryotic community in the rhizosphere, root endosphere, or shoot endosphere, based on PERMANOVA analysis (Table 1). Moreover, there was no clear separation of samples according to plant species either in NMDS ordination (Fig. 2B) or in a heat map investigating the relative abundances of prokaryotic genera across sampled plant species (Fig. 4). It is possible that, rather than employing host-specific mechanisms of microbial selection, which can benefit individual plant species during competition (42), the plants colonizing the disturbed site generally selected microbes that alleviated the contaminant-associated stress induced by the original presence of PHCs. For instance, Oliveira et al. (27) demonstrated such a phenomenon in plants growing in PHC-contaminated soils, which selected endophytes that were able to degrade PHCs. In addition, several studies showed that distantly related plant species growing at different sites in cold regions harbor similar microbial communities: a so-called “cold-adapted plant microbiome” characterized by psychrotolerance and low host-species specialization (43–45). Taking that into consideration, it could also be that a combination of a high concentration of PHCs and the cold climate created a selective pressure that reduced the pool of microorganisms in the soil that were able to associate with the colonizing plants, and hence, little to no effect of colonizing plant species on community composition occurred.

The significant influence of the initial phytoremediation strategy (−P, +P, or +2P) on the diversity and structure of prokaryotic communities associated with colonizing host plants (Table 1; Fig. 3, 5, and 6; Fig. S1) may be indirectly attributed to plant-driven processes that alter physical and chemical attributes of soil, i.e., rhizodeposition (46). Rhizodeposition has been found to play an important role in shaping soil microorganisms that utilize plant-derived compounds as sources of carbon and/or energy (23, 47, 48). The rate of microbial degradation of such compounds depends on their chemical structure and availability in soil and can be influenced by the presence of pollutants. Alternatively, plant compounds were shown to affect the degradation of pollutants (49). Moreover, certain pools of soil organic matter can take years or decades to be transformed by microorganisms (50). Therefore, even after the disappearance of the originally planted grasses (+P and +2P), the initial input of organic carbon to PHC-contaminated soils through rhizodeposition and subsequent plant litter deposition likely shaped prokaryotic communities associated with the next generations of plant successors. In addition, microbial communities associated with the planted grasses (Lolium multiflorum and Festuca rubra) could have persisted in PHC-contaminated soils to later inhabit the colonizing plants, which could explain the significant association of the prokaryotic communities with the phytoremediation strategies found herein (Table 1; Fig. 5 and 6). Several recent studies indicated that soil legacy, including the microorganisms originating from past vegetation, influences the succession of the next generations of plants by altering the germination rate and plant growth (51–53). We thus hypothesize that the transfer of potentially beneficial microorganisms among different generations of plants could be important in contaminated soils, including those in extreme environments. Such a process could aid in establishing and maintaining biodiversity at previously disturbed sites, especially in the case of cold-adapted plant microbiota with a wide spectrum of host specificity. Here, for instance, ASVs belonging to the plant growth-promoting diazotrophic genera Bradyrhizobium, Mesorhizobium, and Allorhizobium-Neorhizobium-Pararhizobium-Rhizobium were found to be differentially abundant among phytoremediation treatments. While rhizobia may have contributed to the distinct patterns of plant and microbial succession in these nutrient-poor subarctic soils previously contaminated with crude oil and diesel, testing such hypotheses is beyond the scope of this study and requires further research.

Our analyses indicate that each bioremediation strategy (−P, +P, and +2P) led to the significant enrichment of specific bacterial ASVs associated with colonizing plants. Accordingly, as there was a significant interaction between the original soil type and the phytoremediation strategy (Table 1), the enriched ASVs were distinct for crude- and diesel-contaminated soils (Fig. 5 and 6). Several of these enriched ASVs belonged to genera that were previously reported to contain members that degrade PHCs; for instance, Flavobacterium, Bacillus, Pseudomonas, Corynebacterium, Staphylococcus, Burkholderia, Kocuria, Micrococcus, and Streptococcus (16, 54). Furthermore, such enrichment of specific ASVs likely corresponds to the original presence of either crude oil or diesel. It was shown that different sources of PHCs varying in their chemical compositions were associated with different microbial communities, including PHC-degrading populations (55–58). It should be noted, though, that the soil texture also varied between the two contaminated soils (12), which may have contributed to different patterns of microbial succession as well. Overall, our results suggest that the originally planted grasses had either served as a source of some of the facultative endophytes that had persisted in PHC-contaminated soils and/or provided an environment favorable to these specific ASVs that later became associated with the colonizing vegetation that succeeded in crude oil- and/or diesel-contaminated soils.

Finally, fertilization has previously been shown to stimulate the bioremediation of polluted soils through the enhancement of plant growth and microbial degradation of pollutants (59–62). Such effects have been observed in PHC-contaminated subarctic soils upon the addition of a fertilizer (12, 63). In this study, the interaction of two factors implemented at the beginning of the original study, the application of mineral fertilizer and the original soil type, was still significantly associated with the prokaryotic communities in the rhizosphere and roots of plants growing at the site almost 20 years later. Taken together, the significant association of fertilization, original soil type, and phytoremediation strategy with prokaryotic communities of colonizing plants further points toward the long-lasting impacts that a single treatment can have on plant-associated microbiota in subarctic environments previously disturbed by PHC contamination.

To conclude, we present novel information that extends the understanding of microbial succession in soil and plants in cold regions. By using high-throughput amplicon sequencing, we were able to characterize the structure and diversity of plant-associated prokaryotic communities following initial phytoremediation treatment of PHC-contaminated soils, using different grass species (Lolium multiflorum and Festuca rubra) as phytoremediation agents. Even after almost 20 years and the disappearance of the initially planted grasses, the interaction of the original soil type and phytoremediation strategy, rather than the current host plant, were among the drivers of prokaryotic communities associated with plants that had successfully colonized the site. Furthermore, each phytoremediation strategy led to the significant enrichment of distinct bacterial ASVs in the rhizosphere and endospheres of colonizing plants, which may have initially helped the colonizing plants to survive and adapt to PHC-contaminated soils. Nevertheless, the reader should be once again reminded that the original treatments influenced patterns of secondary succession at the research site (12). Thereby, it is possible that, even though we did not find a strong association between the colonizing plant species and plant-associated microbial community composition, the legacy of the original treatments detected in the rhizosphere, roots, and shoots of volunteer vegetation might be, at least partially, a secondary effect of distinct secondary succession strategies that occurred at the site. Overall, the outcome of this study provides evidence that in previously disturbed subarctic ecosystems, the non-host-specific soil-to-root transfer of microorganisms seems to be of great importance for the succession of plant-associated microbiota. Future research might therefore aim to decipher how different environmental factors promote either horizontal or vertical transmission of microorganisms among different generations and species of plants in subarctic regions. Finally, the results of our study highlight the role of phytoremediation not only in the removal of PHCs but also as a legacy that determines the long-term microbial succession of soils previously contaminated by PHCs. Understanding the microbial assemblages associated with vegetation colonizing restored soils is valuable for the management of contaminated subarctic soils with the goals of sustainably maintaining ecosystem function and resilience.

## MATERIALS AND METHODS

### Experimental site and sampling.

The original phytoremediation experiment was established at the Farmers Loop Permafrost Research Facility field site of the Army Corps of Engineers Cold Regions Research and Engineering Laboratory (ACE CRREL) located in Fairbanks, Alaska, in 1995. The soils used in the experiment included crude oil-contaminated soil collected from a gravel pad at a pump station on the Trans-Alaska pipeline and diesel-contaminated soil collected during the removal of an underground storage tank. In addition to the difference in the original source of PHCs (crude oil or diesel), the soils differed in structure: the crude oil-contaminated soil was gravel with a large grain size, while the diesel-contaminated soil was finer in texture with more organic matter (12). For that reason, the crude oil- and diesel-contaminated soils are referred to herein as the original soil types. The total initial PHC concentrations were approximately 3,420 and 800 mg/kg in crude oil-contaminated soil and diesel-contaminated soil, respectively. Each soil type was separately placed in a lined and bermed area approximately 21 by 3 m and 60 cm deep. Each area was later subdivided into seven treatment plots. The treatments included three levels of vegetation and two levels of nutrient addition for each soil type, as follows: the plots were sown with annual ryegrass (Lolium multiflorum) (+P) or a 1:1 mixture of annual ryegrass and Arctared fescue (Festuca rubra) (+2P) or left unplanted (−P) and were additionally treated with commercially available mineral fertilizer, which was surface applied at approximately 620 g/m^2^ of N, P, and K (granular 20-20-10) (+F), or left unfertilized (−F) (3, 12, 31, 33).

Soil and plant sampling for this study was carried out in September of 2014, by which point the PHC concentrations were below detection limits (<0.5 ppm; EPA method 8015M) in all treatment plots (64). The plots had been successfully colonized by a variety of volunteer herbaceous and woody plants depending on the original phytoremediation strategy (12). The plants (referred to herein as colonizing plants) that were sampled belonged to the following species: Picea glauca (white spruce; WS), Epilobium angustifolium (fireweed; FW), Achillea millefolium (yarrow; Y), Salix spp. (willow; W), Poa spp. (blue grass; L), Populus balsamifera (poplar; P), Shepherdia canadensis (buffalo berry; BB), Betula neoalaskana (birch; B), and Trifolium hybridum (clover; C). Since the site gradually turned from experimental to observational and the experimental plots were not colonized uniformly (12), not all species were harvested from all plots. Sampling was carried out according to the scheme in Fig. 1, with five colonizing plants of five species (i.e., 5 replicas) per plot being harvested.

### Processing of plant and rhizosphere soil samples.

After the removal of bulk soil, the rhizosphere soil samples were collected by shaking off the soil directly adhering to the roots of harvested plants. The roots and shoots were subsequently surface sterilized by immersion in 2% sodium hypochlorite for 15 min. Immediately after surface sterilization, plant samples were rinsed with sterile deionized water three times and finally washed with 10 mM MgSO_4_ to remove sodium hypochlorite. Twenty-microliter amounts of the final wash solutions were spread on plate count agar (PCA) plates and incubated at 25°C for 2 days to confirm sterility. The plant and soil samples were stored at −80°C prior to further analyses.

### DNA extraction and 16S rRNA gene sequencing.

To isolate metagenomic DNA, 0.5 g of the rhizosphere soil and 0.1 to 0.7 g (depending on the quantity harvested) of surface-sterilized roots and shoots were used. Prior to DNA isolation, plant samples were subjected to repeated freezing at −80°C and thawing at 25°C. All plant and rhizosphere soil samples were then homogenized by using the FastDNA spin kit for soil (MP Biomedicals, OH, USA) and the FastPrep instrument for 40 s at 30 m/s followed by 3 min at 15 m/s or for 80 s at 12 m/s, respectively. The DNA was extracted using the same kit following the manufacturer’s instructions and then purified and concentrated using the Genomic DNA Clean and Concentrator-10 kit (Zymo Research Irving, CA, USA).

The metataxonomic analysis of plant-associated prokaryotic communities using 16S rRNA gene amplicon sequencing was done as described previously (14). Briefly, for the soil DNA samples, the 515 forward primer (5′-GTGYCAGCMGCNGCGG-3′; Sigma-Aldrich, USA) and 926 reverse primer (5′-CCGYCAATTYMTTTRAGTTT-3′; Sigma-Aldrich, USA) were used to target the V4-V5 hypervariable region of the 16S rRNA gene (49). The first 15-μL PCR mixture contained 0.02 U/μL Kapa HiFi hot start ready mix (Kapa Biosystems, USA), 0.3 μM each primer (Sigma-Aldrich, USA), 1 μL of template DNA (~15 ng/μL), and PCR-grade water (Sigma-Aldrich, USA). The temperature cycling conditions were as follows: initial denaturation at 95°C for 5 min, 25 to 35 cycles of 20 s at 98°C, 15 s at 56°C, 15 s at 72°C, and final extension at 72°C for 5 min (65). A volume of 0.5 μL of the PCR product was used as a template in a second PCR with the same primers modified with internal barcodes and sequencing adapters of various lengths (49). This round of PCR was performed under the same conditions, but the final reaction mixture volume was 25 μL, the concentration of each primer was 1 mM, and the number of cycles and annealing temperature were decreased to 8 to 14 and 50°C, respectively.

For DNA samples extracted from plant biomass, peptide nucleic acids were used to prevent the amplification of plant organellar DNA (66). The first 15-μL PCR mixture contained 0.3 μM each of peptide nucleic acid probes targeting mitochondrial genes (mPNAs; 5′-GGCAAGTGTTCTTCGGA-3′) and plastid genes (pPNAs; 5′-GGCTCAACCCTGGACAG-3′) (PNA Bio, Thousand Oaks, CA), 0.02 U μL^−1^ of Kapa HiFi hot start ready mix (Kapa Biosystems, USA), 0.3 μM 515 forward primer, 0.3 μM 1068 reverse primer (5′-CTGRCGRCRRCCATGCA-3′; Sigma-Aldrich, USA), 1 μM template DNA (~15 ng), and PCR-grade water (Sigma-Aldrich, USA) (14). The thermal cycling conditions started with a 5-min denaturation at 95°C, followed by 35 cycles at 98°C, 15 s at 72°C (annealing of PNAs), 15 s at 56°C, 15 s at 72°C, and a final extension for 5 min at 72°C. Each sample was prepared in 8 copies that were pooled and separated by electrophoresis on a 1.5% agarose gel. Bands 550 bp in size were excised from the gel and purified using a Zymoclean gel DNA recovery kit (Zymo Research, USA). Then, 0.5 μL of the purified PCR product was used as a template in the same 2-step PCR process as described above for soil DNA samples, except that each round of PCR included the PNAs and an additional step of PNA annealing (15 s at 72°C).

The resulting PCR products were purified using SPRI magnetic beads (Beckman Coulter, USA), and further library preparation and sequencing analysis on an Illumina MiSeq instrument were performed at the Institute of Arctic Biology Genomics Core Laboratory at the University of Alaska Fairbanks, USA. The concentrations of amplicons were normalized to 1 to 2 ng/μL using a SequalPrep kit (Thermo Fisher Scientific, USA), and the samples corresponding to each plate were pooled and either not diluted or diluted 1.5-fold and 3-fold. These three technical replicates of the amplicon libraries were then subjected to 8-cycle PCR to add specific Illumina adapters and sequenced.

### Data analyses.

The sequence data were analyzed using the DADA2 pipeline (67) and R software (version 4.1.0). Several modifications were made to the DADA2 standard operating procedure (SOP) based on the analysis of a mock community, which consisted of 12 bacterial strains (68) that were amplified in parallel with our DNA samples. Briefly, the primer sequences were trimmed off when found present, otherwise the whole read was discarded. To manage the lower sequence quality toward read ends, forward and reverse reads were truncated to lengths of 247 and 170 bp, respectively, and filtered according to their quality using the following parameters: maxN=0, maxEE=1, truncQ=2. After dereplication and the application of DADA2-based removal of sequencing errors, denoised forward and reverse reads were merged and chimeric sequences were removed using the method=“pooled”. Based on the mock community analysis, the sequences differing by one base were clustered and the most abundant sequence was considered the valid one. The technical replicates were merged, and only the sequences that occurred in all technical replicates were kept, resulting in a total number of 6,008,156 reads. Finally, taxonomy was assigned using silva_nr_v132_train_set.fa.gz (69) to create a database of amplicon sequence variants (ASVs). ASVs of plant origin, including mitochondria and chloroplasts, were discarded, which accounted for 12.5% of all sequences. The remaining data set was rarefied to the smallest sample size, 2,300 reads per sample, to ensure the comparability of rhizospheric and endophytic data sets.

Further multivariate statistical analyses of sequence data were conducted using the packages phyloseq (70), vegan (71), DESeq2 (72), ggplot2 (73), and ampvis2 (74). A maximum-likelihood phylogenetic tree (GTR+G+I) was constructed using the packages DECIPHER and phangorn as described by Callahan et al. (75). Prokaryotic diversity was assessed by calculating the Shannon and Simpson alpha diversity indices (75), and the nonparametric Kruskal-Wallis test was used to test whether the prokaryotic diversity differed significantly among plant-associated habitats. To test the statistical significance of the effects of the original soil type (diesel- or crude oil-contaminated soil), fertilization, and phytoremediation strategy on prokaryotic diversity, the Kruskal-Wallis test was performed using Shannon diversity index values calculated separately for the rhizospheric, root endophytic, and shoot endophytic data sets. To investigate the prokaryotic community structures in the rhizosphere, roots, and shoots of colonizing plants, nonmetric multidimensional scaling (NMDS) with weighted UniFrac distances was used. Heat maps were constructed using the package ampvis2 (74). The statistical significance of the effects of the original soil type, fertilization, phytoremediation strategy, and colonizing host plant species on the prokaryotic communities of colonizing plants was determined separately on the rhizospheric, root endophytic, and shoot endophytic data sets of ASVs by permutational multivariate analysis of variance (PERMANOVA) (76) implemented in the adonis2 function of the vegan package (71) and based on Bray-Curtis dissimilarity. For each data set, *R*^2^ values were calculated for all individual variables using the adonis function, and subsequently, the order of the variables in the final PERMANOVA model was sorted in descending order based on their respective *R*^2^ values. Redundancy analysis (RDA) based on centered log ratio-transformed data (77) was then conducted to investigate the association of plant-associated prokaryotic communities with the factors studied (the original soil type, phytoremediation strategy, fertilization, and colonizing host plant species) at the ASV level. To identify prokaryotic ASVs that were significantly enriched among treatments, differential abundance analysis was conducted on the unrarefied data set of ASVs using the Wald test with Benjamini-Hochberg multiple testing correction (*P*_adj_ < 0.01) as implemented in the DESeq2 package (72). The function lfcShrink was used to shrink the log_2_ fold changes.

### Data availability.

The unprocessed FASTQ files for all samples were deposited in SRA under BioProject accession number PRJNA771088.

## References

[1] Schmidt CW. 2012. Offshore exploration to commence in the Arctic: can Shell’s oil-spill response plans keep up? Environ Health Perspect 120:A194–A199. 22548969 10.1289/ehp.120-a194 PMC3346799

[2] Leigh MB, Matz A, Gamberg M. 2019. Contaminants in the northwest boreal region synthesis project—drivers of landscape change. *In* Sesser AL, Rockhill AP, Magness DR, Reid D, Delapp J, Burton PJ, Schroff E, Barber V, Markon C (ed), Drivers of landscape change in the northwest boreal region. University of Alaska Press, Fairbanks, AK.

[3] Reynolds C, Koenen B, Perry L, Pidgeon C. 1997. Initial field results for rhizosphere treatment of contaminated soils in cold regions, p 143–146. *In* Zubeck H, Woolard C, White D, Vinson T (ed), Proceedings of the Fifth International Association of Cold Region Development. American Society of Civil Engineers, Anchorage, AK.

[4] Yergeau E, Lawrence JR, Sanschagrin S, Waiser MJ, Korber DR, Greer CW. 2012. Next-generation sequencing of microbial communities in the Athabasca River and its tributaries in relation to oil sands mining activities. Appl Environ Microbiol 78:7626–7637. doi:10.1128/AEM.02036-12. 22923391 PMC3485728

[5] Mohn WW, Stewart GR. 2000. Limiting factors for hydrocarbon biodegradation at low temperature in Arctic soils. Soil Biol Biochem 32:1161–1172. doi:10.1016/S0038-0717(00)00032-8.

[6] Truskewycz A, Gundry TD, Khudur LS, Kolobaric A, Taha M, Aburto-Medina A, Ball AS, Shahsavari E. 2019. Petroleum hydrocarbon contamination in terrestrial ecosystems—fate and microbial responses. Molecules 24:3400. doi:10.3390/molecules24183400. 31546774 PMC6767264

[7] Yang S, Wen X, Shi Y, Liebner S, Jin H, Perfumo A. 2016. Hydrocarbon degraders establish at the costs of microbial richness, abundance and keystone taxa after crude oil contamination in permafrost environments. Sci Rep 6:37473. doi:10.1038/srep37473. 27886221 PMC5122841

[8] Filler DM, Snape I, Barnes DL. 2008. Bioremediation of petroleum hydrocarbons in cold regions. Cambridge University Press, Cambridge, UK.

[9] Gkorezis P, Daghio M, Franzetti A, Van Hamme JD, Sillen W, Vangronsveld J. 2016. The interaction between plants and bacteria in the remediation of petroleum hydrocarbons: an environmental perspective. Front Microbiol 7:1836. doi:10.3389/fmicb.2016.01836. 27917161 PMC5116465

[10] Macek T, Macková M, Káš J. 2000. Exploitation of plants for the removal of organics in environmental remediation. Biotechnol Adv 18:23–34. doi:10.1016/S0734-9750(99)00034-8. 14538117

[11] Correa-García S, Pande P, Séguin A, St-Arnaud M, Yergeau E. 2018. Rhizoremediation of petroleum hydrocarbons: a model system for plant microbiome manipulation. Microb Biotechnol 11:819–832. doi:10.1111/1751-7915.13303. 30066464 PMC6116750

[12] Leewis M-C, Reynolds CM, Leigh MB. 2013. Long-term effects of nutrient addition and phytoremediation on diesel and crude oil contaminated soils in subarctic Alaska. Cold Reg Sci Technol 96:129–137. doi:10.1016/j.coldregions.2013.08.011. 24501438 PMC3909700

[13] Palmroth MR, Pichtel J, Puhakka JA. 2002. Phytoremediation of subarctic soil contaminated with diesel fuel. Bioresour Technol 84:221–228. doi:10.1016/s0960-8524(02)00055-x. 12118697

[14] Lopez-Echartea E, Strejcek M, Mukherjee S, Uhlik O, Yrjälä K. 2020. Bacterial succession in oil-contaminated soil under phytoremediation with poplars. Chemosphere 243:125242. doi:10.1016/j.chemosphere.2019.125242. 31995861

[15] Leewis M-C, Uhlik O, Fraraccio S, McFarlin K, Kottara A, Glover C, Macek T, Leigh MB. 2016. Differential impacts of willow and mineral fertilizer on bacterial communities and biodegradation in diesel fuel oil-contaminated soil. Front Microbiol 7:837. doi:10.3389/fmicb.2016.00837. 27313574 PMC4889597

[16] Leewis M-C, Uhlik O, Leigh MB. 2016. Synergistic processing of biphenyl and benzoate: carbon flow through the bacterial community in polychlorinated-biphenyl-contaminated soil. Sci Rep 6:1–12. doi:10.1038/srep22145. 26915282 PMC4768254

[17] Walworth J, Pond A, Snape I, Rayner J, Ferguson S, Harvey P. 2007. Nitrogen requirements for maximizing petroleum bioremediation in a sub-Antarctic soil. Cold Reg Sci Technol 48:84–91. doi:10.1016/j.coldregions.2006.07.001.

[18] Brown DM, Bonte M, Gill R, Dawick J, Boogaard PJ. 2017. Heavy hydrocarbon fate and transport in the environment. Q J Eng Geol Hydrogeol 50:333–346. doi:10.1144/qjegh2016-142.

[19] Murygina V, Gaydamaka S, Gladchenko M, Zubaydullin A. 2016. Method of aerobic-anaerobic bioremediation of a raised bog in Western Siberia affected by old oil pollution. A pilot test. Int Biodeterior Biodegradation 114:150–156. doi:10.1016/j.ibiod.2016.06.009.

[20] Coulon F, Brassington KJ, Bazin R, Linnet P, Thomas K, Mitchell T, Lethbridge G, Smith J, Pollard SJ. 2012. Effect of fertilizer formulation and bioaugmentation on biodegradation and leaching of crude oils and refined products in soils. Environ Technol 33:1879–1893. doi:10.1080/09593330.2011.650221. 23240181

[21] Zhang PP, Le Zhang Y, Jia JC, Cui YX, Wang X, Zhang XC, Wang YQ. 2020. Revegetation pattern affecting accumulation of organic carbon and total nitrogen in reclaimed mine soils. PeerJ 8:e8563. doi:10.7717/peerj.8563. 32201638 PMC7071818

[22] Buta M, Blaga G, Paulette L, Păcurar I, Roșca S, Borsai O, Grecu F, Sînziana PE, Negrușier C. 2019. Soil reclamation of abandoned mine lands by revegetation in northwestern part of Transylvania: a 40-year retrospective study. Sustainability 11:3393. doi:10.3390/su11123393.

[23] Philippot L, Raaijmakers JM, Lemanceau P, van der Putten WH. 2013. Going back to the roots: the microbial ecology of the rhizosphere. Nat Rev Microbiol 11:789–799. doi:10.1038/nrmicro3109. 24056930

[24] White JF, Kingsley KL, Zhang Q, Verma R, Obi N, Dvinskikh S, Elmore MT, Verma SK, Gond SK, Kowalski KP. 2019. Review: endophytic microbes and their potential applications in crop management. Pest Manag Sci 75:2558–2565. doi:10.1002/ps.5527. 31228333 PMC6771842

[25] Papik J, Folkmanova M, Polivkova M, Suman J, Uhlik O. 2020. The invisible life inside plants: deciphering the riddles of endophytic bacterial diversity. Biotechnol Adv 44:107614. doi:10.1016/j.biotechadv.2020.107614. 32858117

[26] Hardoim PR, van Overbeek LS, Berg G, Pirttilä AM, Compant S, Campisano A, Döring M, Sessitsch A. 2015. The hidden world within plants: ecological and evolutionary considerations for defining functioning of microbial endophytes. Microbiol Mol Biol Rev 79:293–320. doi:10.1128/MMBR.00050-14. 26136581 PMC4488371

[27] Oliveira V, Gomes NC, Almeida A, Silva AM, Simões MM, Smalla K, Cunha Â. 2014. Hydrocarbon contamination and plant species determine the phylogenetic and functional diversity of endophytic degrading bacteria. Mol Ecol 23:1392–1404. doi:10.1111/mec.12559. 24765659

[28] Zhu X, Ni X, Liu J, Gao Y. 2014. Application of endophytic bacteria to reduce persistent organic pollutants contamination in plants. Clean Soil Air Water 42:306–310. doi:10.1002/clen.201200314.

[29] Acuña-Rodríguez IS, Newsham KK, Gundel PE, Torres-Díaz C, Molina-Montenegro MA. 2020. Functional roles of microbial symbionts in plant cold tolerance. Ecol Lett 23:1034–1048. doi:10.1111/ele.13502. 32281227

[30] Giauque H, Connor EW, Hawkes CV. 2019. Endophyte traits relevant to stress tolerance, resource use and habitat of origin predict effects on host plants. New Phytol 221:2239–2249. doi:10.1111/nph.15504. 30276818

[31] Reynolds C, Koenen B, Carnahan J, Walworth J, Bhunia P. 1997. Rhizosphere and nutrient effects on remediating subarctic soils, p 297–302. *In* Alleman BC, Leeson A (ed), In situ and on-site bioremediation: Papers from the Fourth International In Situ and On-Site Bioremediation Symposium, vol 4. New Orleans, April 28-May 1, 1997. Battelle Memorial Institute, New Orleans, LA.

[32] Reynolds C, Koenen BA. 1997. Rhizosphere-enhanced bioremediation. Mil Eng 89:32–33.

[33] Reynolds C, Wolf D, Gentry T, Perry L, Pidgeon C, Koenen B, Rogers H, Beyrouty C. 1999. Plant enhancement of indigenous soil micro-organisms: a low-cost treatment of contaminated soils. Polar Rec 35:33–40. doi:10.1017/S0032247400026310.

[34] Coleman-Derr D, Desgarennes D, Fonseca-Garcia C, Gross S, Clingenpeel S, Woyke T, North G, Visel A, Partida-Martinez LP, Tringe SG. 2016. Plant compartment and biogeography affect microbiome composition in cultivated and native Agave species. New Phytol 209:798–811. doi:10.1111/nph.13697. 26467257 PMC5057366

[35] Yang R, Liu P, Ye W. 2017. Illumina-based analysis of endophytic bacterial diversity of tree peony (Paeonia Sect. Moutan) roots and leaves. Braz J Microbiol 48:695–705. doi:10.1016/j.bjm.2017.02.009. 28606427 PMC5628320

[36] Polivkova M, Suman J, Strejcek M, Kracmarova M, Hradilova M, Filipova A, Cajthaml T, Macek T, Uhlik O. 2018. Diversity of root-associated microbial populations of Tamarix parviflora cultivated under various conditions. Appl Soil Ecol 125:264–272. doi:10.1016/j.apsoil.2018.02.002.

[37] Lundberg DS, Lebeis SL, Paredes SH, Yourstone S, Gehring J, Malfatti S, Tremblay J, Engelbrektson A, Kunin V, Del Rio TG, Edgar RC, Eickhorst T, Ley RE, Hugenholtz P, Tringe SG, Dangl JL. 2012. Defining the core Arabidopsis thaliana root microbiome. Nature 488:86–90. doi:10.1038/nature11237. 22859206 PMC4074413

[38] Gottel NR, Castro HF, Kerley M, Yang Z, Pelletier DA, Podar M, Karpinets T, Uberbacher E, Tuskan GA, Vilgalys R, Doktycz MJ, Schadt CW. 2011. Distinct microbial communities within the endosphere and rhizosphere of Populus deltoides roots across contrasting soil types. Appl Environ Microbiol 77:5934–5944. doi:10.1128/AEM.05255-11. 21764952 PMC3165402

[39] Müller H, Berg C, Landa BB, Auerbach A, Moissl-Eichinger C, Berg G. 2015. Plant genotype-specific archaeal and bacterial endophytes but similar Bacillus antagonists colonize Mediterranean olive trees. Front Microbiol 6:138. doi:10.3389/fmicb.2015.00138. 25784898 PMC4347506

[40] Albrectsen BR, Siddique AB, Decker VHG, Unterseher M, Robinson KM. 2018. Both plant genotype and herbivory shape aspen endophyte communities. Oecologia 187:535–545. doi:10.1007/s00442-018-4097-3. 29492690 PMC5997111

[41] Ridl J, Kolar M, Strejcek M, Strnad H, Stursa P, Paces J, Macek T, Uhlik O. 2016. Plants rather than mineral fertilization shape microbial community structure and functional potential in legacy contaminated soil. Front Microbiol 7:995. doi:10.3389/fmicb.2016.00995. 27446035 PMC4919359

[42] Hassani MA, Durán P, Hacquard S. 2018. Microbial interactions within the plant holobiont. Microbiome 6:58. doi:10.1186/s40168-018-0445-0. 29587885 PMC5870681

[43] Massoni J, Bortfeld-Miller M, Jardillier L, Salazar G, Sunagawa S, Vorholt JA. 2020. Consistent host and organ occupancy of phyllosphere bacteria in a community of wild herbaceous plant species. ISME J 14:245–258. doi:10.1038/s41396-019-0531-8. 31624344 PMC6908658

[44] Carrell AA, Carper DL, Frank AC. 2016. Subalpine conifers in different geographical locations host highly similar foliar bacterial endophyte communities. FEMS Microbiol Ecol 92:fiw124. doi:10.1093/femsec/fiw124. 27267931

[45] Sheng HM, Gao HS, Xue LG, Ding S, Song CL, Feng HY, An LZ. 2011. Analysis of the composition and characteristics of culturable endophytic bacteria within subnival plants of the Tianshan Mountains, northwestern China. Curr Microbiol 62:923–932. doi:10.1007/s00284-010-9800-5. 21061126

[46] Lynch JM, Whipps JM. 1990. Substrate flow in the rhizosphere. Plant Soil 129:1–10. doi:10.1007/BF00011685.

[47] Tian T, Reverdy A, She Q, Sun B, Chai Y. 2020. The role of rhizodeposits in shaping rhizomicrobiome. Environ Microbiol Rep 12:160–172. doi:10.1111/1758-2229.12816. 31858707

[48] Paterson E, Gebbing T, Abel C, Sim A, Telfer G. 2007. Rhizodeposition shapes rhizosphere microbial community structure in organic soil. New Phytol 173:600–610. doi:10.1111/j.1469-8137.2006.01931.x. 17244055

[49] Fraraccio S, Strejcek M, Dolinova I, Macek T, Uhlik O. 2017. Secondary compound hypothesis revisited: selected plant secondary metabolites promote bacterial degradation of cis-1,2-dichloroethylene (cDCE). Sci Rep 7:1–11. doi:10.1038/s41598-017-07760-1. 28814712 PMC5559444

[50] Dungait JA, Hopkins DW, Gregory AS, Whitmore AP. 2012. Soil organic matter turnover is governed by accessibility not recalcitrance. Glob Change Biol 18:1781–1796. doi:10.1111/j.1365-2486.2012.02665.x.

[51] De Long JR, Heinen R, Jongen R, Hannula SE, Huberty M, Kielak AM, Steinauer K, Bezemer TM. 2021. How plant–soil feedbacks influence the next generation of plants. Ecol Res 36:32–44. doi:10.1111/1440-1703.12165.

[52] Heinen R, Hannula SE, De Long JR, Huberty M, Jongen R, Kielak A, Steinauer K, Zhu F, Bezemer TM. 2020. Plant community composition steers grassland vegetation via soil legacy effects. Ecol Lett 23:973–982. doi:10.1111/ele.13497. 32266749 PMC7318629

[53] Hannula SE, Heinen R, Huberty M, Steinauer K, De Long JR, Jongen R, Bezemer TM. 2021. Persistence of plant-mediated microbial soil legacy effects in soil and inside roots. Nat Commun 12:1–13. doi:10.1038/s41467-021-25971-z. 34584090 PMC8478921

[54] Xu X, Liu W, Tian S, Wang W, Qi Q, Jiang P, Gao X, Li F, Li H, Yu H. 2018. Petroleum hydrocarbon-degrading bacteria for the remediation of oil pollution under aerobic conditions: a perspective analysis. Front Microbiol 9:2885. doi:10.3389/fmicb.2018.02885. 30559725 PMC6287552

[55] Bidja Abena MT, Chen G, Chen Z, Zheng X, Li S, Li T, Zhong W. 2020. Microbial diversity changes and enrichment of potential petroleum hydrocarbon degraders in crude oil-, diesel-, and gasoline-contaminated soil. 3 Biotech 10:42. doi:10.1007/s13205-019-2027-7. PMC6954164 31988836

[56] Morales-Guzmán G, Ferrera-Cerrato R, Rivera-Cruz M, Torres-Bustillos LG, Arteaga-Garibay RI, Mendoza-López MR, Esquivel-Cote R, Alarcón A. 2017. Diesel degradation by emulsifying bacteria isolated from soils polluted with weathered petroleum hydrocarbons. Appl Soil Ecol 121:127–134. doi:10.1016/j.apsoil.2017.10.003.

[57] Sutton NB, Maphosa F, Morillo JA, Abu Al-Soud W, Langenhoff AA, Grotenhuis T, Rijnaarts HH, Smidt H. 2013. Impact of long-term diesel contamination on soil microbial community structure. Appl Environ Microbiol 79:619–630. doi:10.1128/AEM.02747-12. 23144139 PMC3553749

[58] Stauffert M, Cravo-Laureau C, Jézéquel R, Barantal S, Cuny P, Gilbert F, Cagnon C, Militon C, Amouroux D, Mahdaoui F, Bouyssiere B, Stora G, Merlin FX, Duran R. 2013. Impact of oil on bacterial community structure in bioturbated sediments. PLoS One 8:e65347. doi:10.1371/journal.pone.0065347. 23762350 PMC3677869

[59] Qin J, Xiong H, Ma H, Li Z. 2019. Effects of different fertilizers on residues of oxytetracycline and microbial activity in soil. Environ Sci Pollut Res Int 26:161–170. doi:10.1007/s11356-018-3603-9. 30387057

[60] Gabi-Mirela M, Sorin M, Elena D, Marian S. 2018. Greenhouse study on the influence of natural biostimulators and fertilizers on improving bean plants growth and microbial activity in oil-polluted soil. Eurobiotech J 2:209–214. doi:10.2478/ebtj-2018-0051.

[61] Nkereuwem M, Edem I, Fagbola O. 2010. Bioremediation of oil-polluted soils with organomineral fertilizer (OMF) and Mexican sunflower (Tithonia diversifolia). Niger J Agric Food Environ 6:13–20.

[62] Chang Y-S, Chang Y-J, Lin C-T, Lee M-C, Wu C-W, Lai Y-H. 2013. Nitrogen fertilization promotes the phytoremediation of cadmium in Pentas lanceolata. Int Biodeterior 85:709–714. doi:10.1016/j.ibiod.2013.05.021.

[63] Ruberto L, Dias R, Lo Balbo A, Vazquez SC, Hernandez EA, Mac Cormack WP. 2009. Influence of nutrients addition and bioaugmentation on the hydrocarbon biodegradation of a chronically contaminated Antarctic soil. J Appl Microbiol 106:1101–1110. doi:10.1111/j.1365-2672.2008.04073.x. 19191978

[64] Test Methods for Evaluating Solid Waste, Physical/Chemical Methods, EPA publication SW-846, Third Edition, Final Updates I (1993), II (1995), IIA (1994), IIB (1995), III (1997), IIIA (1999), IIIB (2005), IV (2008), and V (2015).

[65] Lopez-Echartea E, Strejcek M, Mateju V, Vosahlova S, Kyclt R, Demnerova K, Uhlik O. 2019. Bioremediation of chlorophenol-contaminated sawmill soil using pilot-scale bioreactors under consecutive anaerobic-aerobic conditions. Chemosphere 227:670–680. doi:10.1016/j.chemosphere.2019.04.036. 31022668

[66] Lundberg DS, Yourstone S, Mieczkowski P, Jones CD, Dangl JL. 2013. Practical innovations for high-throughput amplicon sequencing. Nat Methods 10:999–1002. doi:10.1038/nmeth.2634. 23995388

[67] Callahan B, McMurdie PJ, Rosen MJ, Han AW, Johnson AJA, Holmes SP. 2016. DADA2: high-resolution sample inference from Illumina amplicon data. Nat Methods 13:581–583. doi:10.1038/nmeth.3869. 27214047 PMC4927377

[68] Strejcek M, Smrhova T, Junkova P, Uhlik O. 2018. Whole-cell MALDI-TOF MS versus 16S rRNA gene analysis for identification and dereplication of recurrent bacterial isolates. Front Microbiol 9:1294. doi:10.3389/fmicb.2018.01294. 29971049 PMC6018384

[69] Callahan B. 2018. Silva taxonomic training data formatted for DADA2 (Silva version 132). Zenodo, CERN, Geneva, Switzerland.

[70] McMurdie PJ, Holmes S. 2013. phyloseq: an R package for reproducible interactive analysis and graphics of microbiome census data. PLoS One 8:e61217. doi:10.1371/journal.pone.0061217. 23630581 PMC3632530

[71] Oksanen J, Blanchet F, Friendly M, Kindt R, Legendre P, McGlinn D, Minchin P, O’Hara R, Simpson G, Solymos P. 2019. vegan: community ecology package. R package version 2.5-5.

[72] Love MI, Huber W, Anders S. 2014. Moderated estimation of fold change and dispersion for RNA-seq data with DESeq2. Genome Biol 15:550. doi:10.1186/s13059-014-0550-8. 25516281 PMC4302049

[73] Wickham H. 2016. ggplot2: elegant graphics for data analysis, 2nd ed. Springer Nature, New York, NY.

[74] Andersen KS, Kirkegaard RH, Karst SM, Albertsen M. 2018. ampvis2: an R package to analyse and visualise 16S rRNA amplicon data. BioRxiv doi:10.1101/299537.

[75] Callahan B, Sankaran K, Fukuyama J, McMurdie P, Holmes S. 2016. Bioconductor workflow for microbiome data analysis: from raw reads to community analyses. F1000Res 5:1492. doi:10.12688/f1000research.8986.2. 27508062 PMC4955027

[76] Anderson MJ. 2001. A new method for non-parametric multivariate analysis of variance. Austral Ecol 26:32–46. doi:10.1046/j.1442-9993.2001.01070.x.

[77] Aitchison J, Greenacre M. 2002. Biplots of compositional data. J R Stat Soc 51:375–392. doi:10.1111/1467-9876.00275.

