# Supplementary material for: Legacy Effects of Phytoremediation on Plant-Associated Prokaryotic Communities in Remediated Subarctic Soil Historically Contaminated with Petroleum Hydrocarbons
Source: Microbiol Spectr. 2023 Mar 28;11(2):e04448-22. doi: 10.1128/spectrum.04448-22 (PMC10100700; doi:10.1128/spectrum.04448-22)

Figure S1. Shannon diversity index calculated from 16S rRNA gene ASVs originating from the rhizosphere soil (RH), root endosphere (RE), and shoot endosphere (SE) samples of colonizing plants collected from two different soil types (soil previously contaminated with crude oil or diesel), which were originally subjected to different phytoremediation strategies (planting with annual ryegrass (+P), a mix of annual ryegrass and arctared fescue (+2P), or unplanted control (-P)) and initial fertilization treatment (treated with commercial mineral fertilizer (+F) or left unfertilized (−F)).

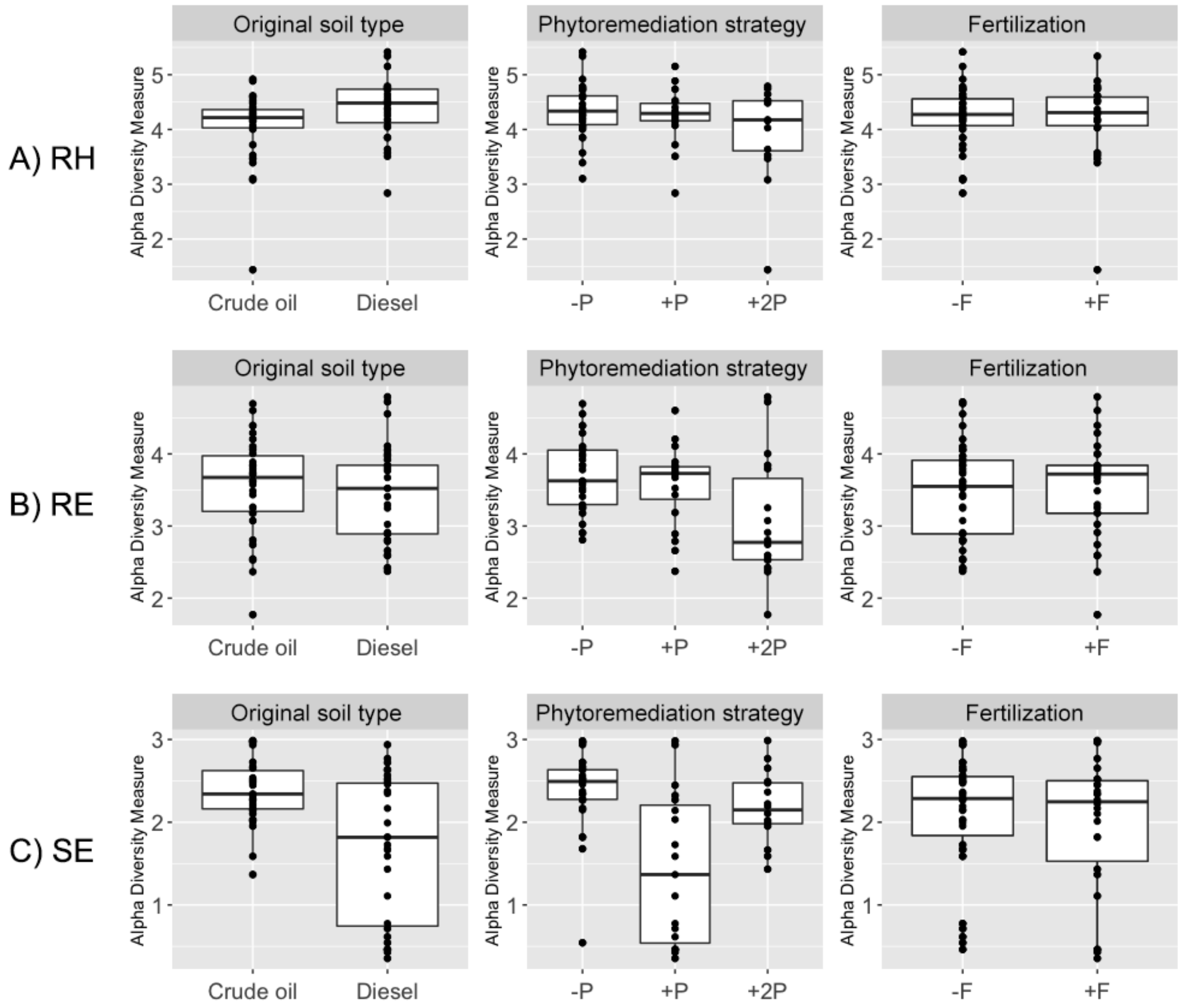

Figure S2. Heatmaps of the 25 most abundant prokaryotic genera in the rhizosphere (RH), root endosphere (RE), and shoot endosphere (SE) samples collected from two different original soil types (soil previously contaminated with crude oil or diesel), which were originally subjected to different phytoremediation strategies (planting with annual ryegrass (+P), a mix of annual ryegrass and arctared fescue (+2P), or unplanted control (-P)) and initial fertilization treatment (treated with commercial mineral fertilizer (+F) or left unfertilized (−F)).

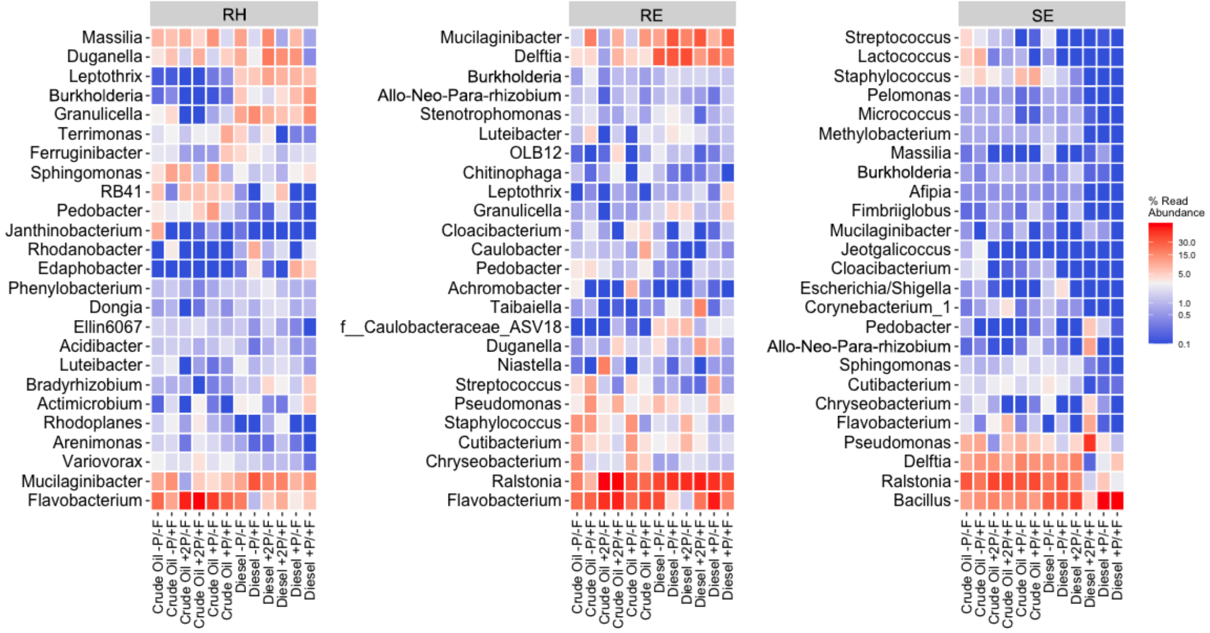

Supplement: Supplemental file 1 — Supplemental material. Download spectrum.04448-22-s0001.pdf, PDF file, 0.3 MB [file spectrum.04448-22-s0001.pdf]
